# Room-Temperature Fabricated Thin-Film Transistors Based on Compounds with Lanthanum and Main Family Element Boron

**DOI:** 10.3390/molecules23061373

**Published:** 2018-06-06

**Authors:** Peng Xiao, Junhua Huang, Ting Dong, Jianing Xie, Jian Yuan, Dongxiang Luo, Baiquan Liu

**Affiliations:** 1 School of Physics and Optoelectronic Engineering, Foshan University, Foshan 528000, China; xiaopeng@fosu.edu.cn (P.X.); jamha1212@163.com (J.H.); xiejianingfs@126.com (J.X.); 2 Guangdong Juhua Printed Display Technology Co. Ltd., Guangzhou 510006, China; dongt@tcl.com; 3 School of Materials and Energy, Guangzhou University of Technology, Guangzhou 510006, China; Luodx@gdut.edu.cn; 4 Institute of Polymer Optoelectronic Materials and Devices, State Key Laboratory of Luminescent Materials and Devices, South China University of Technology, Guangzhou 510640, China; 5 LUMINOUS! Center of Excellence for Semiconductor Lighting and Displays, School of Electrical and Electronic Engineering, Nanyang Technological University, Nanyang Avenue, Singapore 639798, Singapore

**Keywords:** LaB_x_, thin film transistors, low temperature, field effect, flexible

## Abstract

For the first time, compounds with lanthanum from the main family element Boron (LaB_x_) were investigated as an active layer for thin-film transistors (TFTs). Detailed studies showed that the room-temperature fabricated LaB_x_ thin film was in the crystalline state with a relatively narrow optical band gap of 2.28 eV. The atom ration of La/B was related to the working pressure during the sputtering process and the atom ration of La/B increased with the increase of the working pressure, which will result in the freer electrons in the LaB_x_ thin film. LaB_x_-TFT without any intentionally annealing steps exhibited a saturation mobility of 0.44 cm^2^·V^−1^·s^−1^, which is a subthreshold swing (*SS*) of 0.26 V/decade and a *I_on_*/*I_off_* ratio larger than 10^4^. The room-temperature process is attractive for its compatibility with almost all kinds of flexible substrates and the LaB_x_ semiconductor may be a new choice for the channel materials in TFTs.

## 1. Introduction

The flexible active matrix organic light-emitting diode (AMOLED) displays have been attracting great attention because they have many outstanding advantages such as thin width, lightweight, and superior flexibility [1,2,3]. As the key part of the AMOLED, thin film transistors (TFTs) with a low temperature process become an inevitable trend in order to match flexible displays.

Over recent decades, amorphous silicon (a-Si) and polycrystalline silicon have been the main choice for channels in TFTs. However, a-Si TFTs has a low mobility of less than 1 cm^2^·V^−1^·s^−1^, which is too low to drive high resolution displays [4,5]. On the other hand, polycrystalline silicon TFTs possess poor uniformity due to the grain boundary, which limits its application in large-size displays [6,7]. Compared to silicon based TFTs, metal oxide TFTs (e.g., InGaZnO [8,9,10], InZnO [11,12,13], and ZnO [14,15,16]) have a great potential in flat panel displays because of their high mobility, visible-light transparency, satisfactory uniformity, and low temperature process [17,18]. However, TFTs based on metal oxides are meeting a great challenge of long-term stability. Actually, InGaZnO (IGZO) was the most representative among oxide material systems. Since Nomura et al. [19] reported the flexible TFTs based on IGZO, the IGZO has attracted extensive attention. Currently, with the efforts of many scientific researchers, AMOLEDs based on IGZO-TFTs have entered people’s life. However, Indium is a rare earth element and is becoming rarer. Therefore, the cost is very expensive. Furthermore, considering the cost and stability, it is necessary to develop some new materials to fabricate TFTs at a low temperature. To develop In-free materials, Alston et al. [20] reported TFTs with a GaSnZnO (GSZO) active layer fabricated below 150 °C. However, the mobility was only 0.14 cm^2^·V^−1^·s^−1^. Park et al. [21] reported solution-processed TFTs with an alkali metal doped ZnO active layer, but the ZnO surface was sensitive to the atmosphere and the device stability was poor. Kim et al. [22] reported TFTs with an Hf doped ZnO active layer, but the electrical performance was poor with a large subthreshold swing (*SS*) of 1.09 V/decade and a turn-on voltage (*V_on_*) of −7 V. Jiang et al. [23] reported TFTs with an Al doped ZnO active layer, but the mobility was only 0.17 cm^2^·V^−1^·s^−1^. It seems difficult to attain high-performance TFTs with a ZnO based active layer without the Indium element. Therefore, it is necessary to develop a new semiconductor material system suitable for an active layer in TFTs.

Lanthanum hexaboride (LaB_6_) is a known functional ceramic material in the photoelectric field due to its high melting temperature, excellent chemical stability, and high hardness [24,25,26,27]. Furthermore, La is relatively abundant in the earth’s crust with an annual output of 12,500 t compared to the In with an annual output of 75 t and Ga with an annual output of 30 t. In and Ga are limited resources and becoming rarer, so the relatively rich content in the earth’s crust means a lower price. Therefore, LaB_x_ is cheaper than In_2_O_3_ based material for an active layer in TFTs. Considering the physical properties and cost, there is a great potential for LaB_6_ materials in the semiconductor field. Conventionally, LaB_6_ is widely used as cathode emission material [28,29]. So far, there is no report about its application in the TFTs field.

In our work, we report the fabrication and performance of TFTs that use compounds with lanthanum and the main family element Boron (LaB_x_) for the active layer. This is the first time to use LaB_x_ thin film as the channel materials in TFTs. The LaB_x_-TFTs exhibited obvious field-effect characteristics. The structure and performance of LaB_x_ thin films were investigated by X-ray photoelectron spectroscopy (XPS), X-ray diffraction (XRD), and UV-Visible spectrometer. Compared to the TFTs with ZnO-TFTs with the In element, the outstanding advantages of LaB_x_-TFTs include the following: the cost of LaB_6_ is relatively cheap due to the abundant content of La in the earth crust, which is helpful for reducing the manufacturing cost and the stable chemical properties of LaB_6_, which is beneficial for high-stability devices. Additionally, the LaB_6_ has a low coefficient of expansion close to zero, so the stress between LaB_6_ and adjacent films is low and it is easy to attain high-stability flexible devices. Therefore, it may provide a new choice for channel materials in TFTs.

## 2. Experimental

**Figure 1:**
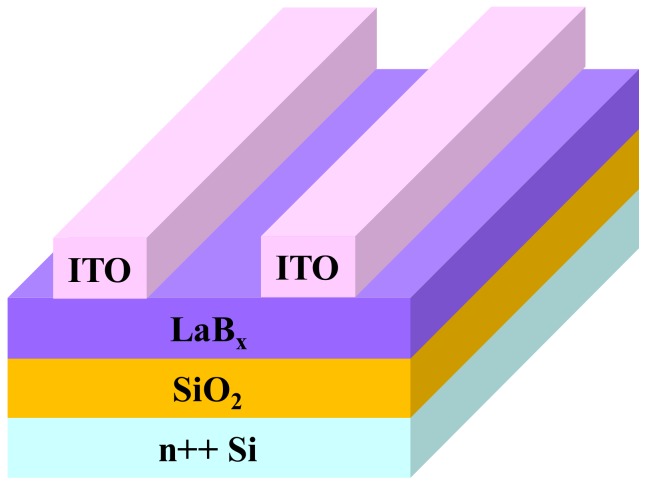
The schematic structure of LaB_x_-TFT.

The LaB_x_-TFTs were fabricated with a top contact configuration (see Figure 1) by using a heavily doped n-type silicon wafer with a 300 nm thick layer of thermally oxidized SiO_2_ (11.4 nF/cm^2^), which serves as the gate electrode and gate insulator, respectively. The wafers were cleaned in an ultrasonic bath with acetone, de-ionized water, detergent, de-ionized water, and isopropanol for 10 min in sequence. The LaB_x_ thin films with a thickness of 40 nm (optimized thickness) were deposited on the silicon wafer by DC magnetron sputtering with LaB_6_ target in a pure argon atmosphere with a flow of 25 sccm and patterned by a shadow mask with an area of 500 μm × 800 μm. For the source/drain electrodes, 380-nm-layer of ITO was sputtered through a shadow mask defining a channel width/length (W/L) of 300/300 μm. The whole preparation process was completed at room temperature. We compared the device A to device B and made a detailed investigation on the different electrical performances between device A and B. In this scenario, the only difference between device A and B is that the LaB_x_ channel layer was prepared under different working pressure. For device A, the LaB_x_ thin film was deposited in pure argon atmosphere with a flow rate of 25 sccm under a working pressure of 0.25 Pa. At the same time, the LaB_x_ thin film was deposited under a working pressure of 3.8 Pa for device B.

## 3. Results and Discussion

### 3.1. Electrical Properties

**Figure 2:**
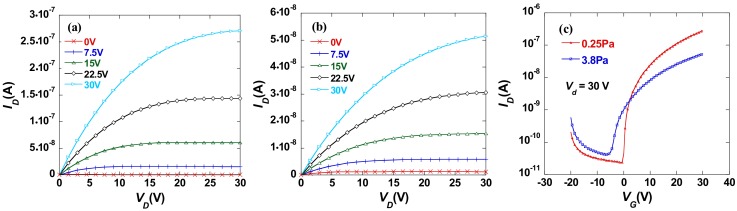
Output curves for device A (**a**) and device B (**b**). (**c**) Transfer curves for device A and device B. (Device A: LaB_x_ active layer was prepared in pure argon atmosphere with a flow rate of 25 sccm under a working pressure of 0.25 Pa. Device B: LaB_x_ active layer was prepared in pure argon atmosphere with a flow rate of 25 sccm under a working pressure of 3.8 Pa).

Figure 2a,b show the output curves for LaB_x_ TFTs fabricated under the different working pressure of 0.25 Pa and 3.8 Pa, respectively. Both device A and B exhibited strongly-saturated output characteristics. Additionally, device A exhibited a larger output current than device B in a saturation region. The comparison between the transfer curves for device A and B is shown in Figure 2c, respectively. The corresponding properties were summarized in Table 1. Device B exhibited a poor electrical performance with a saturation mobility (*μ_sat_*) of 0.13 cm^2^·V^−1^·s^−1^, a subthreshold swing (*SS*) of 0.89 V, a negative turn-on voltage (*V_on_*) of −5.31 V, a negative threshold voltage (*V_T_*) of −2.51 V, and a current on/off (*I_on_*/*I_off_*) larger than 10^3^. At the same time, device A exhibited a relative satisfactory electrical performance with a higher *μ_sat_* of 0.44 cm^2^·V^−1^·s^−1^, a lower *SS* of 0.26 V/decade, a *V_on_* of −0.44 V, a *V_T_*of −2.27 V, and a *I_on_*/*I_off_* ratio larger than 10^4^.

The significant difference between device A and B was mainly ascribed to the different chemical structure and atom ratio for La/B under a different working pressure [30,31]. For LaB_x_ film, the working pressure plays a very important role in the deposition process. Zhao et al. [32] pointed out that LaB_6_ thin films, which were deposited at 1.0 Pa, have a higher degree of crystalline structure and superior physical properties in comparison with the other films. Hu et al. [33] also reported that argon pressure strongly influenced the condensing particles’ kinetic energy clearly by affecting the scattering processes of sputtered energetic particles and LaB_6_ film deposited at 1.0 Pa showed a higher crystallinity degree. However, the optimal conditions are not applicable to LaB_x_ films in this work, which can be used as an active layer for TFTs. It’s noted that the huge difference of atomic weight between La and B is extremely large. For the La atom, the atomic weight is 138.9 while, for the B atom, the atomic weight is only 10.8. This means that the scattering probability of those atoms in discharge space by Ar atoms is very different from each other. The scattering of La atoms is small and La atoms are relatively easy to place at the substrate. On the other hand, B atoms are likely to be scattered by Ar. Therefore, some of them will arrive at the substrate level but some will be deposited at the chamber wall or evacuated by the vacuum pump. This implies that the La/B stoichiometric ratio of LaB_x_ film will be changed when deposited under different working pressures. The structure of LaB_6_ is similar to the that of CsCl, which exhibits a body centered cubic shape [34]. The difference is that the B_6_ octahedral clusters occupy the position of the Cl atom and the La atom occupies the position of the Cs atom. To keep the stability of the B_6_ octahedral network, two electrons are needed. So the La atom with three electrons in the outermost electron orbital will be electronically spared and the extra electron will be free to move around the La atom. In other words, the electrical properties of LaB_x_ thin film will be largely dependent on the chemical structure and the ratio of La and B. It is reasonable to suppose that the free electrons will increase with the increase of the La/B stoichiometric ratios. However, the resistivity (carrier concentration or mobility) is nonlinear with the La/B stoichiometric ratios because it is also affected by the degree of crystallization and the grain boundary scattering in addition to the La/B stoichiometric ratios [33]. To explain the different properties for TFTs with the LaB_x_ active layer prepared under different working pressures, the measurement of XPS, XRD, and UV-visible spectrometer were performed.

### 3.2. XPS Measurement

In order to figure out the composition change of LaB_x_ thin film deposited under different working pressures, the XPS measurement was performed. The 300-nm-thick LaB_x_ thin film samples were prepared on silicon substrate by magnetron sputtering with a LaB_6_ ceramic target. The LaB_x_ thin film samples measured for XRD were prepared under the same conditions. Sample A and sample B denoted for the 300-nm-thick LaB_x_ film were prepared with a working pressure of 0.25 Pa and 3.8 Pa, respectively. In addition, the atomic percentage of each element for sample A and B were summarized in Table 2. As shown in Table 2, there are nearly identical atom percentages of La for sample A (14.0%) and B (15.3%) while a significant difference of atom percentage happened between sample A (49.58%) and sample B (36.9%). The atom ratio of La/B increased from 28.1% to 41.5% with the working pressure increased from 0.25 Pa to 3.8 Pa, which indicates that the relative content of La was increased. This resulted in more free electrons in LaB_x_ thin film. Additionally, this is consistent with the transfer characteristics shown in Figure 2c where the LaB_x_ TFT prepared under the working pressure of 3.8 Pa exhibited a more negative threshold voltage. In addition, the relatively small on-current may be ascribed to the carrier scattering with the increase in carrier concentration.

### 3.3. XRD Patterns

**Figure 3:**
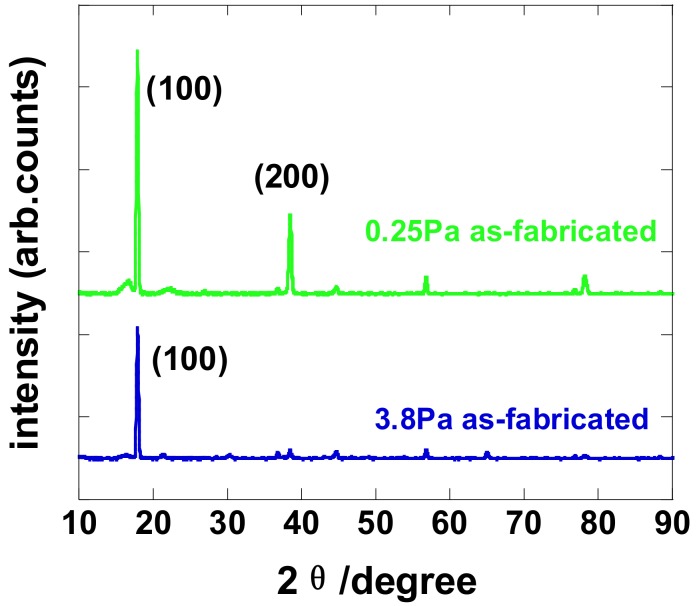
XRD patterns of LaB_x_ thin films prepared under different working pressure. (300 nm on silicon substrate).

The crystal structure of LaB_x_ thin films deposited under different working pressures were investigated by using XRD, which is shown in Figure 3. It is easy to find that the LaB_x_ thin films prepared under different working pressures exhibited obvious crystalline. However, it is noted that we could not match the acquired diffraction patterns to the standard diffraction patterns for LaB_6_. This difficulty can be accounted for by using the following two reasons [35]. First, there is a large thermal mismatch between the LaB_6_ thin film and the Si substrate. The thermal expansion coefficients are 6.0 × 10^−6^ K^−1^ for LaB_6_ versus 2.6 × 10^−6^ K^−1^ for Si and the difference can induce thermal stress in thin films and shift the patterns. Second, due to the Ar implantation, film deposited by sputtering usually has additional problems such as the deviation in the stoichiometric ratio, the defect state’s creation and structural change, which result in the mismatch between the acquired diffraction patterns and reference patterns.

### 3.4. Optical Gap

**Figure 4:**
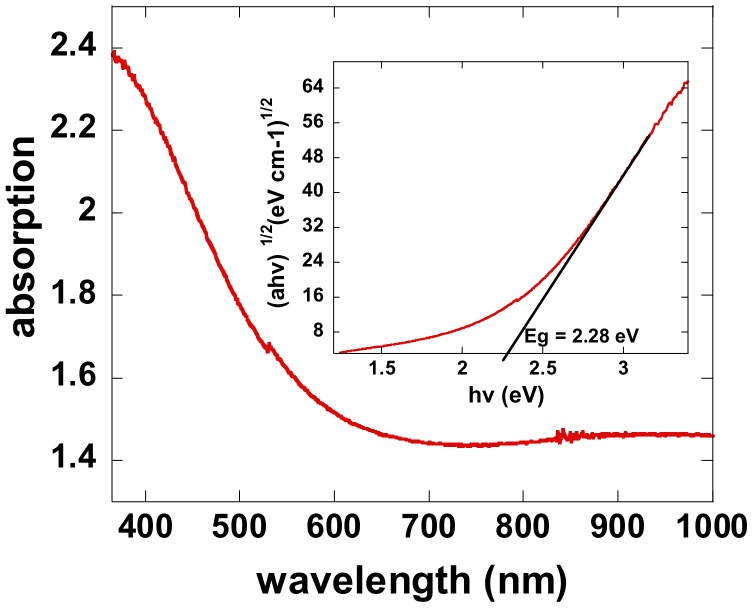
Absorption spectrum of the 40-nm-thick LaB_x_ thin film on quartz glass and the inset shows the plot of (*ahν*)^1/2^ vs. photon energy.

To evaluate the optical bandgap energy (*E_opt_*), the UV-Visible light absorption spectrum was measured. The 40-nm-thick LaB_x_ thin film sample was prepared on quartz glass by magnetron sputtering under a working pressure of 0.25 Pa. The absorption spectrum for LaB_x_ thin film was shown in Figure 4. The Tauc model [36,37] indicates the relationship between the photon energy (*hν*) and the optical-absorption coefficient (*a*). Additionally, the plot of (*ahν*)^1/2^ vs. photon energy was shown in the inset in Figure 4. The *E_opt_* value can be obtained by extrapolating the linear portion to the photon energy axis in the plot of (*ahν*)^1/2^ vs. photon energy. The *E_opt_* value is calculated to be about 2.28 eV. The relatively narrow band gap can lead to smaller activation energies and accumulates the thermally activated carries, which is consistent with the electrical characteristic for LaB_x_ TFT annealed at 400 °C (not shown).

## 4. Conclusions

In conclusion, LaB_x_ thin films prepared under different working pressures by DC magnetron sputtering were investigated as an active layer for TFTs. The element distribution and structural properties of LaB_x_ thin films were analyzed by using XPS, XRD, and UV-visible spectrometers. The XPS results demonstrated that the atom ratio for La/B was related to the working pressure during the sputtering process and enhanced with the increase in the working pressure. The XRD results showed that the LaB_x_ thin film was polycrystalline. According to the absorption spectrum, the *E_opt_* value was calculated to be about 2.28 eV from the plot of (*ahν*)^1/2^ vs. photon energy. The room-temperature fabricated LaB_x_-TFT exhibited a *μ_sat_* of 0.44 cm^2^·V^−1^·s^−1^, a *SS* of 0.26 V/decade, and an *I_on_*/*I_off_* ratio larger than 10^4^. The room-temperature processes without intentionally annealing steps show a great potential for the applications in the flexible displays. The LaB_x_ may be a new choice for the channel materials in TFTs.

## Figures and Tables

**Table 1:** Comparison of device properties for device A and B.

Device Number	*μ*/(cm^2^·V^−1^·s^−1^)	*I_on/off_*	*V_on_*/(V)	*V_T_/*(V)	*s*/(V/decade)
Device A	0.44	1.24 × 10^4^	−0.44	2.27	0.26
Device B	0.13	1.22 × 10^3^	−5.31	−2.51	0.89

**Table 2:** The atomic percentage of each element for the LaB_x_ thin films deposited under different working pressure. (Sample A: a 300-nm LaB_x_ was prepared on silicon substrate by magnetron sputtering with a working pressure of 0.25 Pa. Sample B: a 300-nm LaB_x_ was prepared on silicon substrate by magnetron sputtering with a working pressure of 3.8 Pa).

Sample Number	La/at%	B/at%	O/at%	La/B
Sample A	14.0	49.8	36.2	28.1%
Sample B	15.3	36.9	47.8	41.5%
